# Optical imaging of motor cortical activation using functional near-infrared spectroscopy

**DOI:** 10.1186/1471-2202-13-S1-P27

**Published:** 2012-07-16

**Authors:** Nicoladie D Tam, George Zouridakis

**Affiliations:** 1 Department of Biological Sciences, University of North Texas, Denton, TX 76203, USA; 2 Departments of Engineering Technology, Computer Science, and Electrical and Computer Engineering, University of Houston, Houston, TX, 77204, USA

## 

Functional near-infrared spectroscopy (fNIRS) is an optical imaging technique that allows real-time monitoring of the oxy-hemoglobin (oxy-Hb) and deoxy-hemoglobin (deoxy-Hb) levels in brain tissues in a noninvasive fashion [1,4,6]. The characteristic absorption spectra of oxy-Hb and deoxy-Hb recorded on the scalp can be used to detect the oxygen demands of the underlying brain tissues in the cerebral cortex. This allows real-time detection of brain activation based on metabolic events (neural hemodynamics). Brain imaging using fNIRS has many advantages over fMRI (functional magnetic resonance imaging), as it can detect changes in both oxy- and deoxy-Hb levels simultaneously, whereas an increase in fMRI BOLD (blood-oxygen level dependent) signal only accounts for a decrease in deoxy-Hb levels by an increase in regional cerebral blood volume (rCBV). Additionally, the multi-channel fNIRS signals can be sampled at much higher frequency (in KHz) even though a hemodynamic response may occur at a much slower rate than the sampling frequency. This high temporal resolution is particularly important for capturing dynamic movement activity, such as the high frequencies that result from maximal effort (ME) movements [2]. The latter necessitates the use of fNIRS over fMRI.

The present study uses multi-channel fNIRS to correlate brain activation patterns obtained from the motor cortex with volitional motor execution in healthy human subjects, in an attempt to identify brain-derived command signals that can be used to control a wheelchair. Ultimately, these signals will provide the necessary input to a portable neuroprosthetic device, which will act as a brain-computer interface (BCI) and will enable mobility and navigation for quadriplegics.

In our experiment, subjects were asked to perform directional left-right, forward-backward hand movements while recording fNIRS signals from the motor cortex. Our results show that movement *execution* corresponds to hemodynamic changes seen in both the oxy- and deoxy-Hb signals. The movement is best represented by the summation of oxy- and deoxy-Hb signals, and reflects the total change in hemoglobin concentration (total-Hb), which is a more representative measure of rCBV changes [3] than BOLD rCBV [5]. Intended movement *direction* is correlated with differential changes in these signals, and depends on the specific location of the motor area. Thus, neural activation in the motor cortex can be identified uniquely by the spatiotemporal profiles of localized oxygen delivery, oxygen extraction, and blood flow. These findings provide evidence of the correlation between volitional limb movement direction and motor cortical activation profiles obtained by fNIRS, and support the possibility of a potential BCI neuroprosthetic device for brain-controlled navigation of a wheelchair.

## References

[B1] Calderon-Arnulphi M Alaraj A Slavin KV Near infrared technology in neuroscience: past, present and future Neurol Res 2009 31 6 605 614 10.1179/174313209X383286 19660190

[B2] Colier WNJM Quaresima V Brattelli G Cavallari P van der Sluijs M Ferrari M Detailed evidence of cerebral hemoglobin oxygenation changes in response to motor activation revealed by a continuous wave spectrophotometer with 10 Hz temporal resolution Proc SPIE 1997 2979 390 396

[B3] Deply DT Cope M van der Zee P Aguirre GK Wray S Wyatt J Estimation of optical pathlength through tissue from direct time of flight measurement Phys Med Bio 1998 33 1433 1442 10.1088/0031-9155/33/12/008 3237772

[B4] Hoshi Y Towards the next generation of near-infrared spectroscopy Philos Transact A Math Phys Eng Sci 2011 369 1955 4425 4439 10.1098/rsta.2011.0262 22006899

[B5] Huppert TJ Hoge RD Diamond SG Franceschini MA Boas DA A temporal comparison of BOLD, ASL, and NIRS hemodynamic responses to motor stimuli in adult humans Neuroimage 2006 29 2 368 382 10.1016/j.neuroimage.2005.08.065 16303317 PMC2692693

[B6] sPellicer A Bravo Mdel C Near-infrared spectroscopy: a methodology-focused review Semin Fetal Neonatal Med 2011 16 1 42 49 10.1016/j.siny.2010.05.003 20580625

